# Surface Treatment of Inorganic CsPbI_3_ Nanocrystals with Guanidinium Iodide for Efficient Perovskite Light-Emitting Diodes with High Brightness

**DOI:** 10.1007/s40820-022-00813-9

**Published:** 2022-03-02

**Authors:** Minh Tam Hoang, Amandeep Singh Pannu, Yang Yang, Sepideh Madani, Paul Shaw, Prashant Sonar, Tuquabo Tesfamichael, Hongxia Wang

**Affiliations:** 1 Faculty of Science, School of Chemistry and Physics, Queensland University of Technology, Brisbane, QLD 4001 Australia; 2 Centre for Materials Science, Queensland University of Technology, Brisbane, QLD 4001 Australia; 3 School of Mechanical, Medical and Process Engineering, Faculty of Engineering, Queensland University of Technology, Brisbane, QLD 4001 Australia; 4 Centre for Organic Photonics & Electronics (COPE), School of Chemistry and Molecular Biosciences, The University of Queensland, Brisbane, QLD 4072 Australia

**Keywords:** CsPbI_3_ perovskites, Nanocrystals, Light-emitting diodes, Photoluminescence, Surface passivation, Guanidinium iodide

## Abstract

The remarkable evolution of metal halide perovskites in the past decade makes them promise for next-generation optoelectronic material. In particular, nanocrystals (NCs) of inorganic perovskites have demonstrated excellent performance for light-emitting and display applications. However, the presence of surface defects on the NCs negatively impacts their performance in devices. Herein, we report a compatible facial post-treatment of CsPbI_3_ nanocrystals using guanidinium iodide (GuI). It is found that the GuI treatment effectively passivated the halide vacancy defects on the surface of the NCs while offering effective surface protection and exciton confinement thanks to the beneficial contribution of iodide and guanidinium cation. As a consequence, the film of treated CsPbI_3_ nanocrystals exhibited significantly enhanced luminescence and charge transport properties, leading to high-performance light-emitting diode with maximum external quantum efficiency of 13.8% with high brightness (peak luminance of 7039 cd m^−2^ and a peak current density of 10.8 cd A^−1^). The EQE is over threefold higher than performance of untreated device (EQE: 3.8%). The operational half-lifetime of the treated devices also was significantly improved with T_50_ of 20 min (at current density of 25 mA cm^−2^), outperforming the untreated devices (T_50_ ~ 6 min).

**Supplementary Information:**

The online version contains supplementary material available at 10.1007/s40820-022-00813-9.

## Highlights

- A facile and effective surface passivation strategy was demonstrated to improve the optical and stability of CsPbI_3_ nanocrystals by using guanidinium iodide post-treatment.
- Guanidinium cations was shown to be compatible with CsPbI_3_ perovskite, leading to significantly improved surface properties of CsPbI_3_ nanocrystals.
- Performance of the CsPbI_3_ nanocrystal-based light-emitting device was enhanced by 3.6 folds.

**Supplementary Information:**

The online version contains supplementary material available at 10.1007/s40820-022-00813-9.

## Graphical abstract

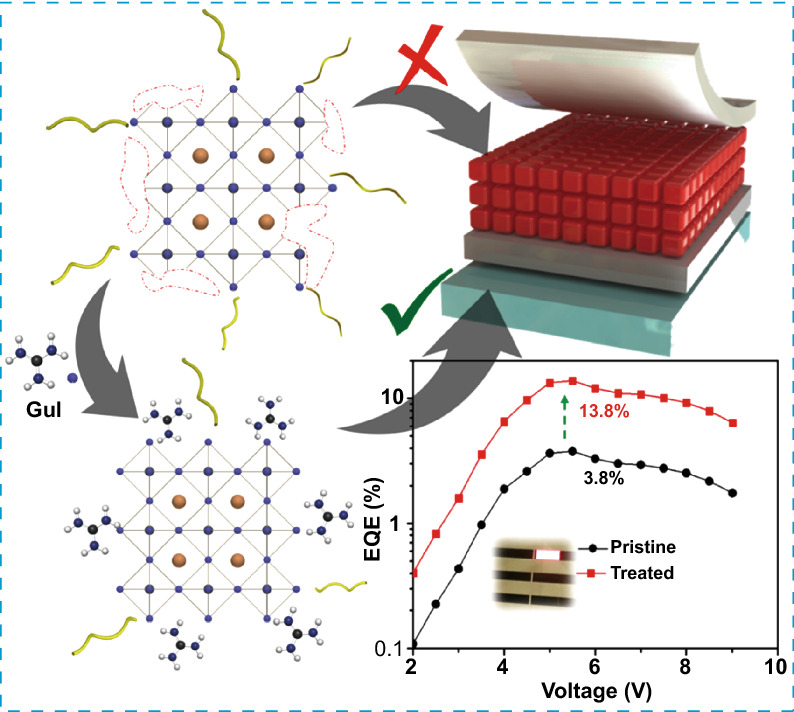

## Introduction

In the past decade, unprecedented progress has been achieved with metal halide perovskites (MHP) in the area of optoelectrical device including solar cells [1], light-emitting diodes (LEDs) [2], and photodetectors [3]. Among the different forms of MHP, nanocrystals (NCs) of MHP are emerging as one of the most exciting optoelectronic materials by virtue of their excellent photophysical properties including tunable, direct bandgap [4], strong quantum confinement [5], high photoluminescence quantum yield [6, 7], and tolerance to defects [8] to name a few. Thanks to the advancement in material engineering of perovskite NCs in terms of morphology, stoichiometric control, doping, and surface treatment [9–14], high-performance light-emitting devices with external quantum efficiency (EQE) of 23.4% and 23.0% have been achieved with MHP NCs for green and red emission, respectively [15, 16]. Nevertheless, most of the peak efficiencies are often observed at relatively low current densities and correspondingly low brightness due to lack of surface protection [17]. There are still several challenging issues in the practical operation of MHP LEDs such as efficiency roll-off (luminescence quenching), ions migration, and stability, which are closely related to their defects [2, 18, 19]. Therefore, methods to effectively suppress the defects and enhance phase stability of the perovskite NCs are of high importance to achieve desirable performance.

Ligands surface stabilization is critical in preserving the phase and optical properties of MHP NCs, thus, the NCs are often synthesized in the presence of high concentration of surface ligands. The ligand density management by using anti-solvent washing after NCs growth is needed to remove the excessive surface ligands and enhance the surface purity. However, the ionic nature of MHPs and labile bonding ligands often lead to a significant number of halide vacancy defects on the surface of perovskite NCs after the purification during synthesis [20]. To address this issue, surface post-treatment strategy using small molecular organic or metal cation has been used to passivate the surface defects and enhance the performance of perovskite NCs [21]. For example, treatment of thin films of perovskite NCs with halide compounds based on AX (where A = formamidinium, methylammonium, or cesium and X = I^−^ or Br^−^) was shown to improve the electronic coupling between the perovskite NCs, enhancing the charge transfer within the NCs film [22]. Co-treatment of 1-hydroxy-3-phenyl propan-2-aminium iodide and tributylsulfonium iodide on CsPbI_3_ NCs enabled the fabrication of LEDs with EQE of 6.4% and stable EL spectra [23]. The recent record of LED-based on CsPbI_3_ NCs with EQE of up to 23% was achieved through surface passivation of CsPbI_3_ NCs with potassium iodide in combination with compositional engineering (Zn^2+^ and Mn^2+^ doping) [16]. However, the device exhibited limited maximum brightness with significant efficiency roll-off observed at 1000 cd m^−2^. There is a wide range of cations which are able to passivate the dangling bonding on surface of MHP NCs. Inorganic cations can provide strong surface coupling but often require using highly polar solvent for dissolution, which can potentially dissolve components of the perovskite NCs or induce defective surface [16, 24]. Fundamentally, a desirable cation for perovskite NCs passivation should (1) enable strong electrical coupling between the perovskite NCs, (2) preserve the quantum confinement of the perovskite NCs and (3) should not adversely alter the structure of the core NCs.

Among the different cations, guanidinium (Gu^+^) is a highly stable small organic cation due to the efficient resonance stabilization with three amino groups [25]. Research shows that treatment of MAPbI_3_ film with guanidinium iodide (GuI) can effectively passivate the grain boundaries of the material surface without altering the crystal structure of the perovskite [26]. A theoretical calculation study has shown that Gu^+^ can simultaneously stabilize the undercoordinated sites at the surface and improve the confinement of charge carriers inside the FAPbBr_3_ nanocrystals thanks to its extra hydrogen bonds from the amino group, significantly benefiting the fabrication of high perovskite FAPbBr_3_ green LED [15]. Nevertheless, the application of Gu^+^ cation on more challenging subjects including red and blue-emitting LED or particularly inorganic perovskite has not yet been investigated.

Herein, we demonstrate a facile surface passivation strategy for inorganic cubic CsPbI_3_ NCs by using guanidinium iodide. Photoluminescence spectroscopy and surface analysis show that the GuI post-treatment of CsPbI_3_ NCs effectively passivates the iodide vacancy defects, significantly enhancing surface stability and confinement of charge carrier in the NCs. Our results also show that the Gu^+^ preferentially resides on the surface of the NCs without changing the perovskite crystal structure or causing NCs coarsening. The treated CsPbI_3_ NCs were employed to make LEDs device with an EQE of 13.8% for red emission (696.5 nm). This is 3.6 times higher than the performance of the untreated devices (EQE: 3.8%). As a result of efficient charge injection, LEDs with treated perovskite NCs also exhibited high brightness with peak luminance of 7039 cd m^−2^, a peak current density of 10.8 cd A^−1^. The operating half-life time T_50_ of the GuI treated device was 20 min at current density of 25 mA cm^−2^, which is also superior to the untreated device under the same condition.

## Experimental Section

### Materials

Cs_2_CO_3_ (99.95%, Sigma Aldrich), Oleic Acid ($\ge$ 99%, Sigma Aldrich), Octadecene (Sigma Aldrich), PbI_2_ (99.99%, Sigma Aldrich), Guanidinium iodide (> 99%, Sigma Aldrich), Oleylamine (Sigma Aldrich), Methyl acetate (MeAc), Hexane (Sigma Aldrich), Tetrahydrofuran (Sigma Aldrich), poly(3,4-ethylenedioxythiophene) polystyrene sulfonate [PEDOT:PSS] (Heraeus), 2,2′,2″-(1,3,5-Benzinetriyl)-tris(1-phenyl-1-H-benzimidazole [TPBI] (Ossila), lithium fluoride [LiF] (Sigma Aldrich), and Silver [Ag] (Sigma Aldrich)**.** The chemicals were used as received without further purification.

### Synthesis of Perovskite NCs

The synthesis procedures were carried out following a published method in literature with some modifications [27]. Cs-Oleate was prepared by adding 202.8 mg of Cs_2_CO_3_ and 10 mL Octadecene (ODE) into 100 mL three-neck flask. The flask was then degassed and dried under vacuum at 120 °C for 30 min. Subsequently, 0.63 mL of Oleic acid (OA) was also added into the solution and the system is degassed for another 30 min. After that, the mixture was heated to 160 °C under N_2_ gas and stir until all the Cs_2_CO_3_ was dissolved to make a clear solution. The Cs-oleate solution is kept at 100 °C under N_2_ gas for further use.

In another three-neck flask, 0.3 mg of PbI_2_ and 20 mL ODE was loaded. The flask also was degassed and dried under vacuum for 1 h at 120 °C. A mixture of 1.5 mL OA and 1.5 mL oleylamine (OLA) was added into the flask during this time. After PbI_2_ was completely dissolved, the temperature was raised to 170 °C and was kept stirring in N_2_ gas for 20 min. At this state, 1.5 mL of as-prepared Cs-Oleate (preheated at 100 °C) was quickly injected into the PbI_2_ solution. About 5 s later, the reaction was quenched in an ice-bath. After the flask is cooled down to room temperature, 25 mL of methyl acetate is added to precipitate the NCs. The NCs are then collected by centrifugation at 10,000 rpm for 10 min. After that, the supernatant is discarded and the collected NCs are dispersed in 10 mL of hexane. Then the NCs solution is washed one more time by adding 10 mL of MeAC and the NCs are recollected by centrifugation at 8000 rpm for 5 min. The collected precipitates are dispersed in 10 mL hexane and keep at 4 °C for 48 h. After this stage, the unreacted substance is precipitated and is remove by centrifugation at 4000 rpm for 5 min. The final supernatant is kept in vacuum for 12 h to remove the residual solvents. The final NCs were dispersed in Hexane for further measurement and applications.

### Guanidinium Post Treatment

The as-synthesized CsPbI_3_ NCs were first dispersed in Hexane (20 mg mL^−1^). 18.7 mg GuI is dissolved in 1 mL THF to make a 0.1 M solution. Different amount of GuI solution was added to 5 mL CsPbI_3_ NCs solution and stir for 5 min. After that, the NCs were recollected by centrifuging at 8000 rpm for 10 min. Supernatant was discarded, precipitate was collected and dispersed into hexane for further use.

### Light Emitting Diode Fabrication

LED devices using pristine and GuI treated CsPbI_3_ NCs as an active light-emitting layer were fabricated using pre-patterned ITO (Xianyan Technology) deposited glass substrates. Before fabrication, the ITO substrates were repeatedly washed with Alconox and deionized water. Further, the ITO substrates were rinsed several times in deionized water before ultra-sonicating them in acetone, ethanol, and isopropanol consecutively for 10 min each. The ITO substrates were blow-dried with compressed air, and UV-plasma treated for 15 min before spin-coating PEDOT: PSS (Heraeus). PEDOT: PSS was filtered by 0.45 μm PVDF filter, before spin coating at 5000 rpm for 30 s using a spin coater (Laurell Technologies). The spin-coated PEDOT: PSS film was removed from contact pads and then annealed at 150 °C for 15 min, before transferring it to a glove box system with low moisture and oxygen (O_2_ < 0.1 ppm, H_2_O < 0.1 ppm). The emitter layers (CsPbI_3_ NCs) solution was prepared in toluene (30 mg mL^−1^) and magnetically stirred for 24 h inside a glovebox. The two-step optimized dynamic spin coating condition was used in a controllable manner over the PEDOT: PSS layer to get a uniform layer of NCs. The emitter layer was dried at room temperature and no further annealing was done. After emitting layer deposition, samples were transferred to another glove box fitted with a torpedo thermal evaporator, without breaking the vacuum. The 40 nm of 2,2′,2″-(1,3,5-Benzinetriyl)-tris(1-phenyl-1-H-benzimidazole [ TPBI] (Ossila), 2 nm of lithium fluoride [LiF] (Sigma Aldrich), and 100 nm of Silver [Ag] (Sigma Aldrich) layers were thermally deposited consequently at pressures ~ 10^–6^ mbar. Each substrate consists of six pixels having an area of each device around 10 mm^2^.

Fabrication of electron-only device: The electron-only device was ITO/SnO_2_/Perovskite NCs/ TPBi/LiF/Ag. The SnO_2_ solution was prepared by diluting a solution of SnO_2_ (15 wt% in H_2_O colloidal dispersion) in DI H_2_O to the concentration of 2.5 wt%. The SnO_2_ solution then was spin-coated on top of ITO substrate at 3000 rpm for 30 s, following by annealing at 180 °C for 30 min.

### Characterization

For characterization, the following measurements were conducted to reveal structure, the optical, electrical properties of the synthesized materials: UV-visible absorbance spectrum of the samples is measured by a UV-visible spectrometer (carry 60) and photoluminescence spectrum was recorded with a Cary Eclipse fluorescence spectrophotometer. The transmission electron microscopy (TEM) images were captured by JEOL 2100 microscope operated at 200 kV. STEM-EDS was conducted using EDS detector coupled with JEOL 2100 TEM. A Kratos AXIS Supra photoelectron spectrometer (He I radiation, hν = 21.22 eV) was used to measure XPS spectra and UPS energy state of the material. A Rigaku Smartlab using monochromatic CuKα (λ = 0.154 nm) as a radiation source is taken to measure the X-ray diffraction (XRD) pattern of the sample. Fourier transform infrared (FTIR) spectroscopy was done by a Bruker model Alpha-P FTIR with ATR accessory. Time-resolved photoluminescence (TRPL) was measured by an Edinburgh fluorescence spectrometer at room temperature. The photoexcited source is a 474 nm wavelength laser with pulse of 82.4 ps. TGA measurement was conducted on a NETZSCH STA 449 F3 Jupiter thermal analyzer with Platinum furnace.

## Results and Discussion

The CsPbI_3_ perovskite NCs were synthesized using hot injection method involving a rapid injection of Cs-oleate solution into the solution of PbI_2_ and ligands (oleic acid and oleylamine) in octadecene at high temperature (170 °C). After this, the NCs were washed with methyl acetate (MeAc) to remove excess ligand and unreacted precursors as advised in previous reports [28, 29].

We verified the effects of MeAC antisolvent used in the washing on the properties of as-synthesized NCs by photoluminescence (PL) and FTIR measurement. The results show that the MeAC anti-solvent washing significantly deteriorated the optical properties of the NCs as evidenced in a reduced PL intensity even though the polarity of methyl acetate is relatively low. Accordingly, the photoluminescence quantum yield (PLQY) dropped from 73.0 to 59.2% after purification (Table S1). The decrease in PLQY is usually observed when the density of ligands on NCs surface is reduced [13]. This is also confirmed in the FTIR measurement (Fig. S1). The CsPbI_3_ NCs without MeAc washing exhibited clear vibration peaks of surface ligands of oleic acid and oleylamine. However, after being washed with MeAc, the intensity of these characteristic peaks is reduced significantly, implying a decrease in the ligand density [13].

By considering that the removal of ligands can leave behind defects and dangling bonds on the surface of the CsPbI_3_ NCs, we developed a strategy using GuI as the post-surface treatment agent. In this approach, we first made a solution of GuI in tetrahydrofuran (THF), which was subsequently added to the dispersion solution of the as-synthesized CsPbI_3_ NCs. Experimental details are included in the supporting information. We used the solution-phase ligand exchange strategy instead of solid state-ligand exchange (treat thin film of NCs) to reduce the effect of particles fusion and coarsening, which is not favorable for light-emitting applications [16, 30]. THF was used as the solvent for dissolving GuI due to its moderately low polarity. It means a small amount of the solvent should not significantly affect the crystal structure of the highly ionic crystals of CsPbI_3_ perovskite.

Interestingly, we found that the emission of the CsPbI_3_ NCs solution was enhanced significantly after the GuI solution was added (Fig. S2). Investigation of the evolution in the PL of the CsPbI_3_ NCs as a function of the volume of GuI solution added showed that the maximum PL emission was achieved with 20 $\mathrm{\mu L}$ of GuI solution added (Fig. S3). Beyond this, the PL dropped dramatically, which is possibly due to the formation of a guanidinium containing non-perovskite phase as reported previously [15, 31]. Furthermore, we observed the appearance of a precipitate as the amount of GuI solution added was increased. We ascribe this to the excessive ligand exchange of oleylammonium with Gu^+^, resulting in the fusion of the NCs to form large particles [32].

**Fig. 1:**
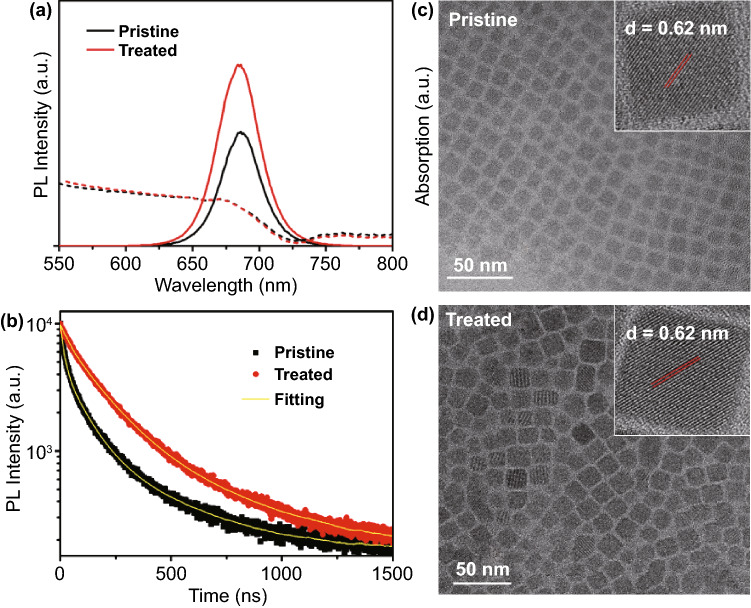
**a** Comparison of UV–visible absorbance (dash line) and photoluminescence (solid line) spectra of pristine CsPbI_3_ NCs and GuI treated CsPbI_3_ NCs. **b** TRPL lifetime measurement of pristine and treated CsPbI_3_ NCs. **c–d** TEM image of the pristine CsPbI_3_ NCs and GuI treated CsPbI_3_ NCs showing their morphology (inset: the HRTEM of individual NCs showing lattice spacing)

Figure 1a shows the PL and UV-vis absorbance of the pristine (0 ${\mu L}$) and treated NCs (20 ${\mu L}$). It can be seen that the GuI treatment does not change the bandgap of the material, as the onset absorption wavelength and PL peak of the pristine and treated samples are in the same position. Both samples show the PL emission peak located at 686 nm. There is only small increase in the full width at half maximum (FWHM) of the PL peak from 34.2 to 36.3 nm after surface treatment, which can be assigned to larger size distribution of the NCs. Relative PLQYs of the NCs solution were determined by using Rhodamine 6G as the reference standard [33]. Detailed calculations for the PLQY values are shown in Table S1. The results show that the GuI treatment enhance the PLQY of the CsPbI_3_ NCs from 59 to 82% with the concentration of NCs solution in the PLQY measurement was approximately 4.5 × 10^–5^ mg mL^−1^. We performed time-resolved PL (TRPL) measurements to determine the PL lifetime of the pristine and GuI treated NCs (Fig. 1b). The parameter obtained from the TRPL fitting are shown in Table S2. The pristine perovskite shows a much faster decay compared to the GuI treated sample. Interestingly, the PL decay of the pristine sample needs to be fitted using a triexponential function rather than a biexponential function. The three-lifetime components of the pristine sample can be associated with Shockley–Read–Hall (SRH) recombination via defect trappings, radiative recombination of free electrons/holes, and Auger recombination [34, 35]. The lifetime decay components $\tau_{1}$, $\tau_{2}$, and $\tau_{3}$ in the pristine sample were 3.34, 19.82, and 76.75 ns, respectively. While for the GuI treated CsPbI_3_ NCs, the PL lifetime decay can readily be fitted with a biexponential function where the fast decay $\tau_{1}$ (25.42 ns) and long decay $\tau_{2}$ (85.4 ns) components can be associated with radiative recombination process [35]. Clearly, there is significant number of traps associated defects presented in the pristine CsPbI_3_ NCs sample, which are successfully filled with GuI treatment. Most importantly, the GuI treated sample exhibits a much longer average lifetime (72.6 ns comparing to 64.7 ns in the pristine NCs), indicating improved quality of the perovskite NCs for efficient radiative recombination. This is probably because the GuI treatment efficiently suppressed the defects related charge trapping and promoted the radiative recombination in the CsPbI_3_ NCs.

The morphology of the perovskite NCs revealed by TEM (Fig. 1c–d) shows that the pristine CsPbI_3_ NCs exhibit a uniform cubic shape with an average size of ~ 11.8 nm. The high-resolution TEM measurement reveals a lattice spacing of 0.62 nm of the NCs, corresponding to the (100) plane of cubic phase CsPbI_3_ perovskite [27, 36]. The energy dispersive X-ray (EDX) elemental mapping shows clear distribution of Cs, Pb, and I over the NCs (Fig. S4). Upon GuI treatment, there is a slight increase in size of the particles from ~ 11.8 to ~ 14.0 nm. However, the NCs still maintained a uniform cubic shape with good crystallinity (Fig. 1d). The change in morphology of the treated NCs can be assigned to the dissolution and coarsening of NCs during ligands exchange process, which is commonly observed in perovskite NCs after surface treatment [37, 38]. Nevertheless, comparing to the solid-sate ligand exchange strategy, the effect of particle fusion and coarsening is less significant when this solution-phase ligand exchange was used. We have conducted SEM measurements to reveal the morphology of CsPbI_3_ NCs film prepared by using different ligand exchange strategy (Fig. S5). The result shows that both pristine CsPbI_3_ NCs film and the solution-phase treated CsPbI_3_ NCs film exhibited a uniform morphology with well-distributed small particles. While the CsPbI_3_ NCs films made by post-treatment with solid-state ligand exchange of GuI (0.5 mg mL^−1^ in ethyl acetate) have more compact morphology with closer packs of particles due to the reduced interdot interaction, which is consistent with the observation in other works using solid-state ligand exchange strategy [30, 32].

**Fig. 2:**
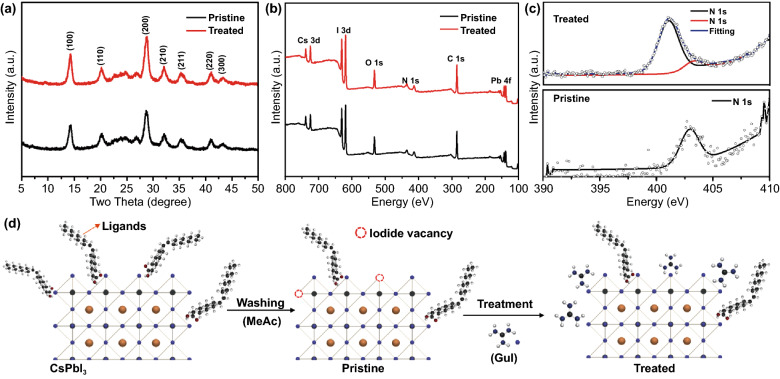
**a** XRD patterns of pristine and treated CsPbI_3_ NCs. **b** Wide XPS spectra and **c** high-resolution N 1s XPS spectra of pristine and treated NCs. **d** Illustration of the passivation process on surface of CsPbI_3_ NCs using GuI treatment

The XRD measurement was carried out to confirm the crystal structure of the as-synthesized NCs. As shown in Fig. 2a, both the pristine and the treated CsPbI_3_ NCs show similar XRD pattern, which belongs to cubic phase CsPbI_3_ (PDF 01-080-4039) with a preferential orientation along the (100) and (200) planes. No distinguishable XRD peak shift was detected with the GuI treated sample, implying that the GuI treatment does not alter the crystal structure of the CsPbI_3_ NCs. In other words, the Gu^+^ cation does not enter the crystal lattice of CsPbI_3_. The result is consistent with previous study on the Gu^+^ treatment of thin film of CsPbI_3_ perovskite [39].

X-ray photoelectron spectroscopy (XPS) was performed to characterize the chemical state of element on the NCs surface. The characteristic XPS signal for Cs 3d, Pb 4f, I 3d of CsPbI_3_ and O 1s, C 1s, N 1s associated with ligand bonding were detected as shown in the XPS scan spectrum of pristine and treated samples **(**Fig. 2b). A closer analysis of the high-resolution XPS spectrum of Cs 3d, Pb 4f, and I 3d shows that the GuI treatment does not affect the chemical state and bonding of these elements in CsPbI_3_ as all the peaks appear in similar shape and position (Fig. S6), which agree with the XRD result that the Gu^+^ cation does not incorporate into the lattice of CsPbI_3_ crystal. On the other hand, there is a noticeable change in the N 1s peak related to the surface ligands. In the pristine NCs, the N 1s peak can be fitted with a single peak at 402.8 eV, which corresponds to the protonated amine groups from oleyl ammonium ligand [40]. The treated NCs show a much stronger N 1s signal, which can be fitted with two peaks at 403.0 and 401.2 eV as shown in Fig. 2c**.** The N 1s peak at 403.0 eV can be assigned to the amine groups in oleyl ammonium similar to pristine NCs. The dominating N 1s peak at 401.2 eV is probably originates from the deprotonated guanidinium group with three amino groups [41]. This result implies the existence of Gu^+^ on the NCs surface, which provides extra amino group to help passivate the CsPbI_3_ NCs surface. This is consistent with previous research [15]. The quantitative analysis of the XPS spectra shows that there was a change in the relative atomic content of elements. Specifically, the atomic ratio of I/Pb was 2.64 in the pristine NCs. This ratio increased to 2.92 in the GuI treated NCs, suggesting that GuI treatment compensates the iodide loss in the washing step, heals the iodide vacancy on the surface of pristine CsPbI_3_ NCs. The result is consistent with the reduced defects confirmed in the above TRPL result. Furthermore, the significant increase of the integrated area of N 1s in the treated sample and increased atomic ratio of N/Pb from 0.18 (pristine sample) to 0.31 (treated sample) also confirm the presence of extra amino related to guanidinium cations on the surface of the NCs.

We further used the thermogravimetric analysis (TGA) to investigate the decomposition of organic ligands in the NCs (Fig. S7). For the pristine sample, a significant weight loss (~ 11 wt%) was observed when the temperature reach to roughly 200 °C, which corresponds to the loss of OA and OLA ligands [42]. The loss above 400 °C can be assigned to the thermal decomposition of CsPbI_3_ perovskite [43]. In contrast, the weight loss from OA and OLA ligand in treated NCs at 200 °C was significantly smaller (3 wt%). In addition, there is a sharp drop at around 300 °C which can be assigned to the melting of guanidinium iodide [44]. This implies that the Gu^+^ cation has been exchanged with the native OA and OLA ligand on the NCs.

Based on these findings, we propose the mechanism underlying the GuI post-treatment as illustrated in Fig. 2d. The CsPbI_3_ NCs have less ligands on the surface after being washed by methyl acetate (MeAc), resulting in exposure of halide vacancies on the surface which can act as charge carrier traps [45]. The GuI treatment provides an iodide source to fill the iodide vacancies on the perovskite NCs surface. Meanwhile, the Gu^+^ cation strongly couples to the surface of the NCs through extra hydrogen bond to further protect the NCs from aggregation. Considering the larger size of Gu^+^ (278 pm) comparing to the size of Cs^+^ cation (177 pm), Gu^+^ will preferentially locate to the surface of CsPbI_3_ instead of entering inside the crystal [15, 46], forming a passivation layer on the surface of CsPbI_3_ which can enhance the excitons confinement. This explains the enhanced PLQY (Fig. 1a).

**Fig. 3:**
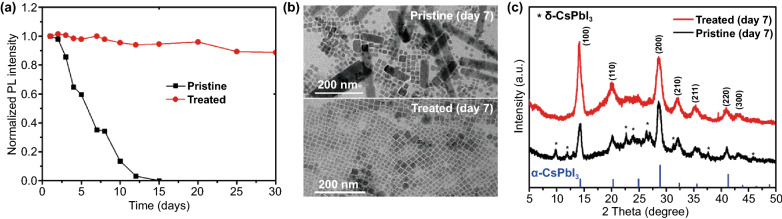
**a** Recorded photoluminescence of pristine and GuI treated CsPbI_3_ NCs solution during storage in ambient condition. **b–c** TEM images and XRD pattern of the corresponding NCs showing the morphology and crystallinity change during storage

We also measured the stability of the NCs by recording the PL emission of the NCs solution during storage in ambient air. The CsPbI_3_ NCs generally show a tendency to agglomerate due to the high surface energy of the small particles, making perovskite NCs solution unstable during storage [47]. In particular, when the ligand density on the surface is low after antisolvent treatment, the NCs often show degradation within 1 week of storage in ambient air [40]. As expected, the pristine CsPbI_3_ solution exhibited quite poor stability in ambient condition (relative humidity 40–65%). The PL of the solution was almost completely quenched after 10 days of storage. In contrast, the treated NCs exhibited no significant change after the first week and still maintained ~ 90% of the original PL emission even after 30 days (Fig. 3a). We see that the untreated NCs after aging time (7 days) have aggregated and fused into large particle and rods with hundreds of nanometre in size (Fig. 3b), which commonly occurs in CsPbI_3_ NCs after prolonged storage [48]. The XRD measurement also confirms the phase transformation of NCs from cubic α-CsPbI_3_ to orthorhombic δ-CsPbI_3_ (Fig. 3c). This explains the loss in photoluminescence. Clearly, the GuI treated NCs exhibit much-enhanced morphology and crystallinity stability. Under the same storage condition, the treated NCs remain dispersed cubic particles and retain their α-CsPbI_3_ phase (Fig. 3b–c). The enhanced stability can be ascribed to the passivating effect of the guanidinium cations on the surface of the NCs and the reduction of halide vacancy defects.

**Fig. 4:**
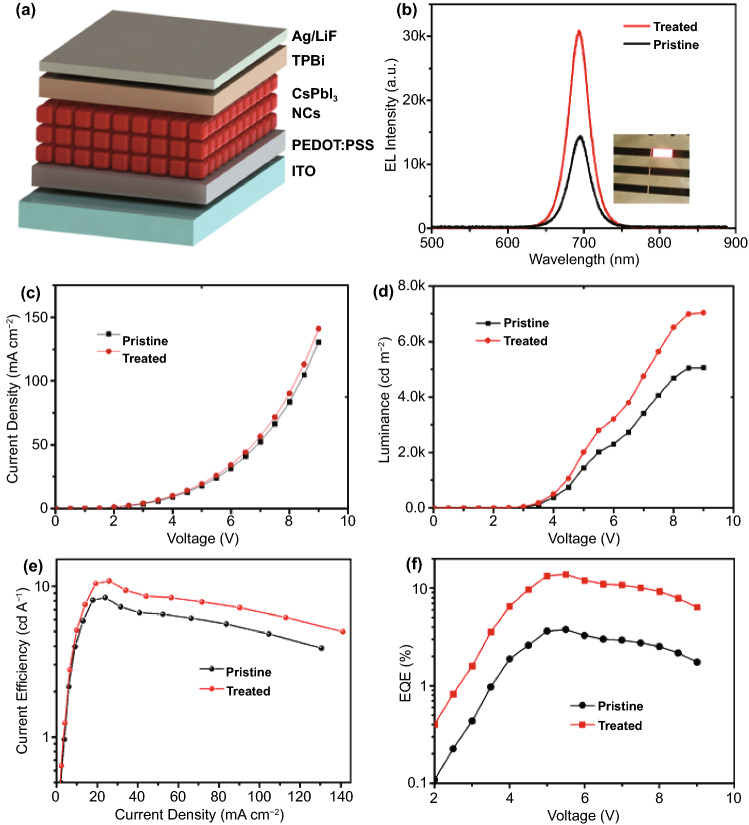
**a** Illustration of CsPbI_3_ NCs LEDs device configuration. **b** Electroluminescence of the LEDs device made from pristine and treated CsPbI_3_ NCs (inset is the picture of LED device emitting red light). **c** Current density–voltage (J–V), **d** luminance–voltage (L–V), **e** current efficiency–current density, and (**f**) external quantum efficiency of the fabricated LED devices from pristine and treated CsPbI_3_ NCs

To demonstrate the benefit of GuI treated CsPbI_3_ in optoelectronic applications, we fabricated LED devices by using the pristine and treated CsPbI_3_ NCs respectively. The device configuration was indium tin oxide electrode/ poly(3,4-ethylene dioxythiophene):poly styrene sulfonate (PEDOT:PSS) (hole injection layer)/ CsPbI_3_ NCs (emissive layer)/ 2,2′,2″-(1,3,5-benzinetriyl)-tris(1-phenyl-1-H-benzimidazole) (TPBi) (electron injection layer) and Ag/LiF (counter electrode) as illustrated in Fig. 4a. Ultraviolet photoelectron spectroscopy (UPS) was used to measure the valance band of the NCs film. It shows that the GuI treatment slightly affects the electronic band structure of CsPbI_3_ NCs (Fig. S8) which can be due to the change in the electronic environment on the surface of NCs after ligand exchange. The band energy alignment of the materials in the device is illustrated in Fig. S8c. The LED devices exhibited stable and uniform electroluminescence (EL) (Fig. 4b-inset). Comparison of the EL spectra (Fig. 4b**)** shows that both devices show the same EL peak position at 695 nm and narrow full width at half maximum of 31.2 nm for the pristine and 32.0 nm for the treated NCs, implying high colour purity. The EL spectrum corresponds to the Commission Internationale del’Eclairage (CIE) color coordinates of (0.62, 0.28) (Fig. S9). There is a slight red shift of the EL peak comparing to the PL peak of the NCs solution in hexane. This is explained by the dielectric dispersion of the solvent and the interdot interaction in the thin films of NCs [49, 50]. At different driving voltage, the EL spectra of the devices remain stable with no significant spectral shift was detected (Fig. S10). It also can be seen that at the same applied voltage, the treated device exhibited higher EL emission (Fig. 4b). The I–V (current–voltage) characteristic in Fig. 4c shows that the treated device exhibits slight enhancement in the current density over the applied voltage range, which agrees with the result that GuI treatment does not alter the electronic structure of CsPbI_3_ NCs. However, the treated device exhibited much-improved luminance, implying higher current efficiency. The maximum luminance up to 7039 cd m^−2^ was achieved with the treated LED device at 9 V applied voltage (Fig. 4d), while the luminance of pristine device only reached to 5064 cd m^−2^. The reproducibility of the luminance of multiple devices (25 devices) is shown in histogram of maximum luminance in Fig. S11, indicating high reproducibility. Both the devices also show low turn-on voltage (at luminance of 1 cd m^−2^) of only ~ 2 V which is near to the optical bandgap (~ 1.72 eV refer Fig. S8) of the material. Further, there is significant improvement obtained in the current efficiency of the treated device (Fig. 4e) which shows a maximum current efficiency of 10.8 cd A^−1^ compared to 8.4 cd A^−1^ of the pristine device. The LEDs device with treated NCs showed a high EQE of 13.8%, which is ~ 3.6 time higher than device based on pristine CsPbI_3_ NCs (3.8%) (Fig. 4f).

**Fig. 5:**
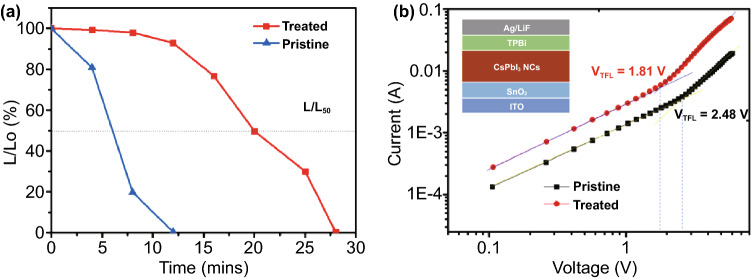
**a** The graph showing the stability of LED devices fabricate from the pristine and GuI treated CsPbI_3_ NCs. The device was operated at constant current density of 25 mA cm^−2^. **b** The logarithm I–V curves of the electron-only devices fabricated from treated and pristine CsPbI_3_ NCs with determined V_TFL_. The inset is the configuration of the device

We also have measured the operational stability of the device (Fig. 5a). The device was operated at constant current density of 25 mA cm^−2^ and the luminance of the device was recorded. The device fabricated by using the pristine CsPbI_3_ exhibited a much faster drop in the luminance with the half lifetime T_50_ was only around 6 min. While the device fabricated with the GuI treated CsPbI_3_ showed much-enhanced stability with the T_50_ ~ 20 min was achieved. The result clearly demonstrated the beneficial effect of GuI treatment method on performance of CsPbI_3_ NCs based LED devices. The performance of our devices is competing among the perovskite NCs LEDs employing different surface treatment method reported previously (Table S3). The results prove the beneficial effect of GuI treatment on enhancing the radiative recombination in the CsPbI_3_ emission layer.

In order to investigate the charge carrier mobility and trap density in the as-fabricated CsPbI_3_ film, we conducted the Space-charge-limited current (SCLC) measurement on the electron-only device (Fig. 5b). An obvious increase in the injection current of the device with treated CsPbI_3_ NCs comparing to the device fabricated with pristine CsPbI_3_ NCs. The result indicates a higher electron injection efficiency [13]. In addition, the device with treated NCs exhibited a trap-filling limited voltage (V_TFL_) of 1.81 V, which is significantly lower than that of the devices fabricated with pristine NCs (2.48 V). The reduction in V_TFL_ obviously was resulted from the lower trap state density in the GuI treated CsPbI_3_ NC film, reflecting the effectiveness of this surface treatment method.

## Conclusions

In conclusion, we successfully demonstrated a compatible surface treatment for inorganic CsPbI_3_ perovskite nanocrystal by using guanidinium iodide. As the effect of antisolvent purification on CsPbI_3_ NCs is severe in term of defects formation, the treatment provides a halide-rich condition to compensate the iodide deficiency in CsPbI_3_ NCs. At the same time, inheriting the excellent surface passivation property of guanidinium cation with extra amino groups, the treatment led to a much-enhanced luminescence property and stability of the CsPbI_3_ NCs. As a result, the LEDs device fabricated with GuI treated CsPbI_3_ achieved effective EQE of 13.8%, luminance of 7039 cd m^−2^ and current efficiency of 10.8 cd A^−1^, which are significantly enhanced comparing to the device made with pristine CsPbI_3_ NCs (EQE: 3.8%; luminance: 5064 cd m^−2^; current efficiency: 8.4 cd A^−1^). This work highlights the importance and prospect of surface defect suppression in achieving higher performance perovskite-based optoelectronic device.

## Supplementary Information

Below is the link to the electronic supplementary material.

Supplementary file1 (PDF 999 KB)
