# Supplementary material for: Surface Treatment of Inorganic CsPbI3 Nanocrystals with Guanidinium Iodide for Efficient Perovskite Light-Emitting Diodes with High Brightness
Source: Nanomicro Lett. 2022 Mar 2;14:69. doi: 10.1007/s40820-022-00813-9 (PMC8891416; doi:10.1007/s40820-022-00813-9)
Supplement: Supplementary file 1 — Supplementary file1 (PDF 999 KB) [file 40820_2022_813_MOESM1_ESM.pdf]

Supporting Information for

Surface Treatment of Inorganic $CsPbI_3$ Nanocrystals with Guanidinium Iodide for Efficient Perovskite Light Emitting Diodes with High Brightness

Minh Tam Hoang[1, 2, ‡], Amandeep Singh Pannu[1, 2 ‡], Yang Yang[1, 2], Sepideh Madani[1, 2], Paul Shaw[4], Prashant Sonar[1, 2], Tuquabo Tesfamichael[2, 3] and Hongxia Wang[1, 2, *]

[1] School of Chemistry and Physics, Faculty of Science, Queensland University of Technology, Brisbane, QLD 4001, Australia

[2] Centre for Materials Science, Queensland University of Technology, Brisbane, QLD 4001, Australia

[3] School of Mechanical, Medical and Process Engineering, Faculty of Engineering, Queensland University of Technology, Brisbane, QLD 4001, Australia

[4] Centre for Organic Photonics & Electronics (COPE), School of Chemistry and Molecular Biosciences, The University of Queensland, Brisbane 4072, Australia

[‡]Minh Tam Hoang and Amandeep Singh Pannu contributed equally to this work.

*Corresponding author. E-mail: hx.wang@qut.edu.au (Hongxia Wang)

Supplementary Figures and Tables

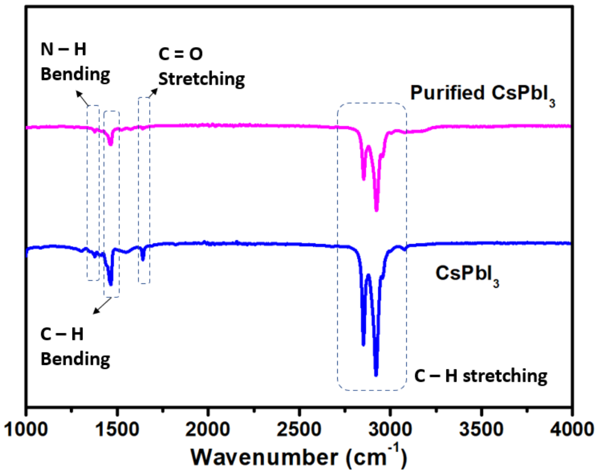

Fig. S1 FTIR spectra of the $CsPbI_3$ NCs before and after purification with Hexane/methyl acetate ligand washing

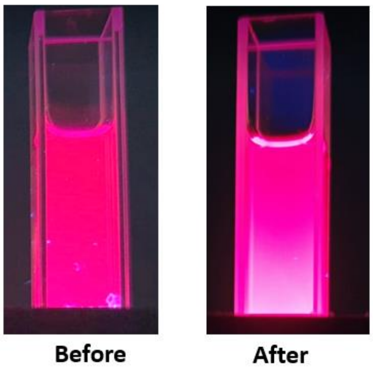

Fig. S2 The picture of solution of $CsPbI_3$ NCs in hexane before and after guanidinium iodide treatment. The solution is emitting red light under UV-365nm excitation

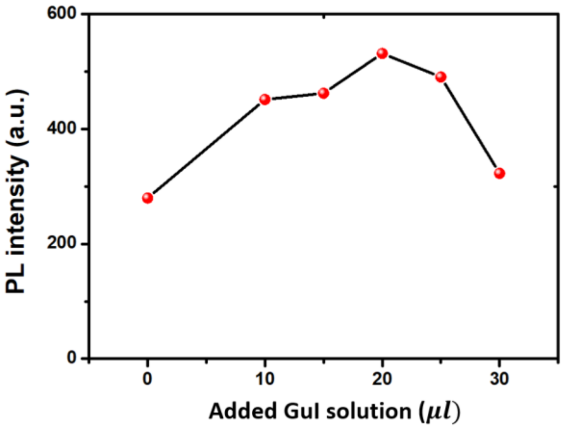

Fig. S3 The evolution of PL emission of $CsPbI_3$ solution with different volume of GuI solution added in the post treatment step

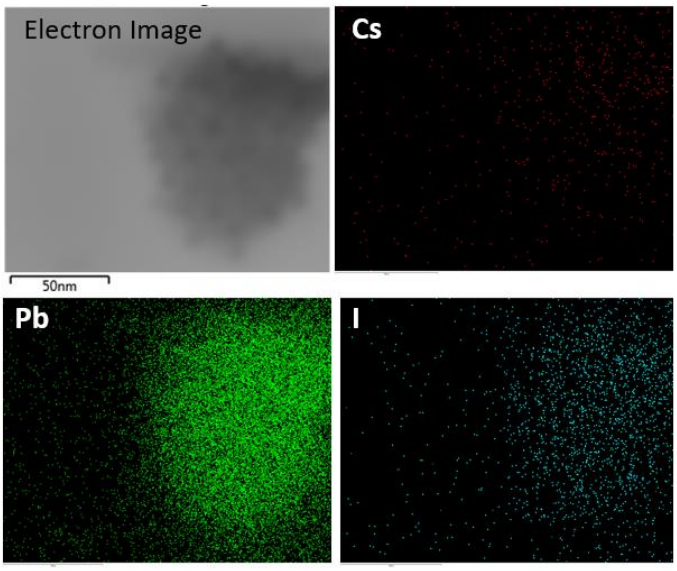

Fig. S4 The energy dispersive X-ray (EDX) elemental mapping of $CsPbI_3$ NCs showing clear distribution of Cs, Pb and I elements

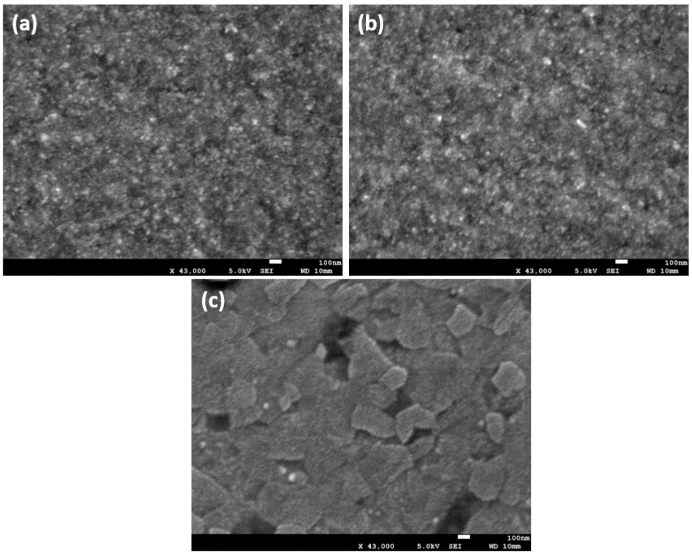

Fig. S5 The SEM images showing the morphology of (**a**) $CsPbI_3$ NCs film, (**b**) solution-phase GuI treated $CsPbI_3$ film and (**c**) $CsPbI_3$ NCs film after solid-state ligand exchange treatment with GuI solution (0.5 mg/ml in ethyl acetate)

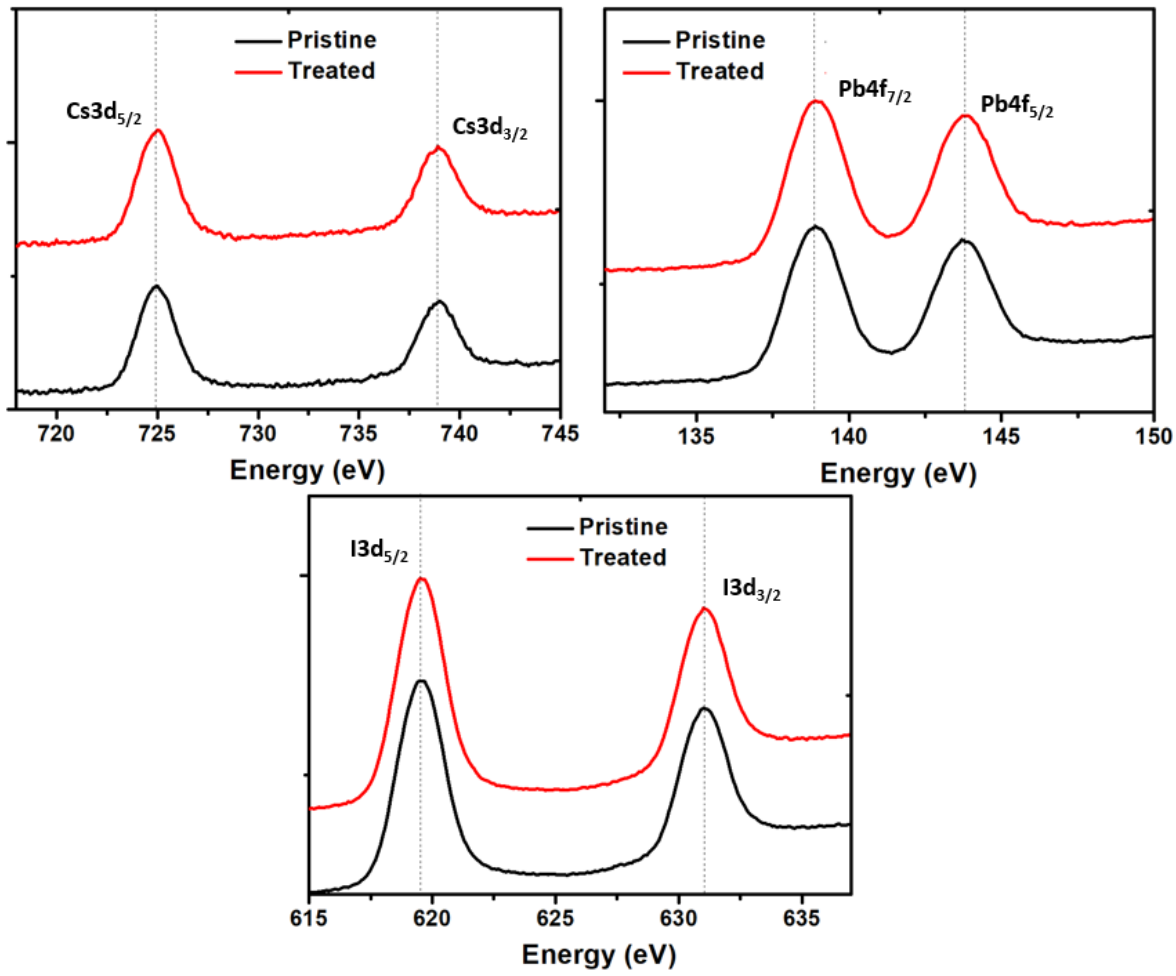

Fig. S6 High resolution XPS of showing Cs 3d, Pb 4f and I 3d signal of pristine $CsPbI_3$ NCs in comparison with GuI treated $CsPbI_3$ NCs

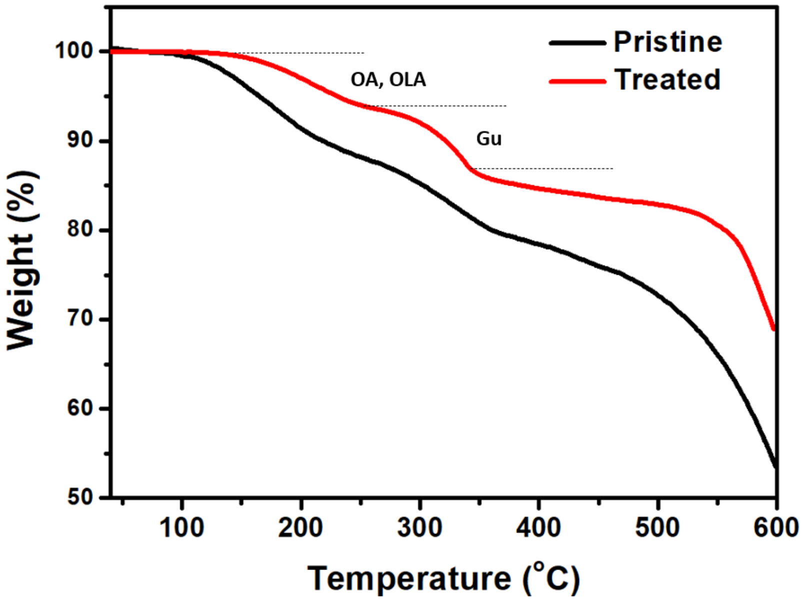

Fig. S7 TGA measurement showing the thermal decomposition of the pristine and GuI treated $CsPBI_3$ NCs

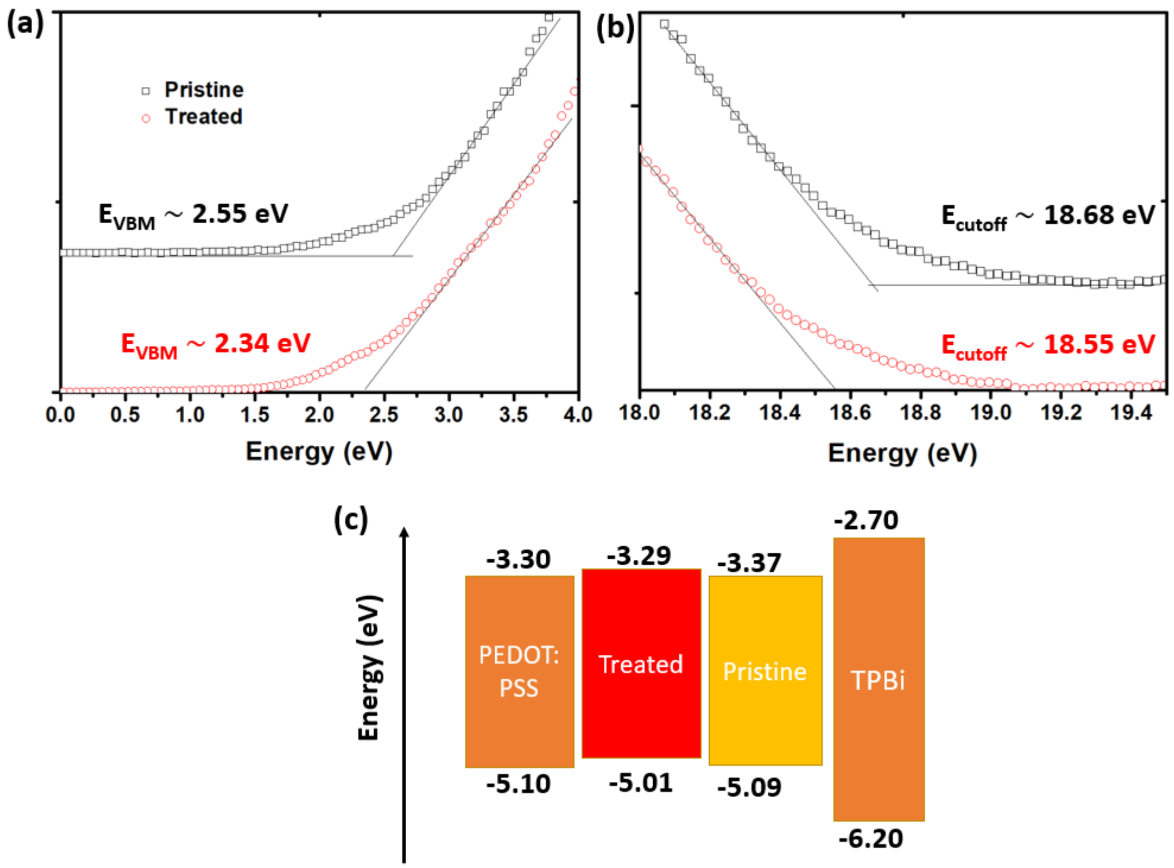

Fig. S8 (a-b) UPS measurement of pristine and treated $CsPbI_3$ NCs, the graph showing the valance band maximum energy and the cut off energy. The black line is the fitting line.; (**c**) Illustration of energy band alignment of the pristine and treated $CsPbI_3$ NCs in between of PEDOT:PSS and TPBi

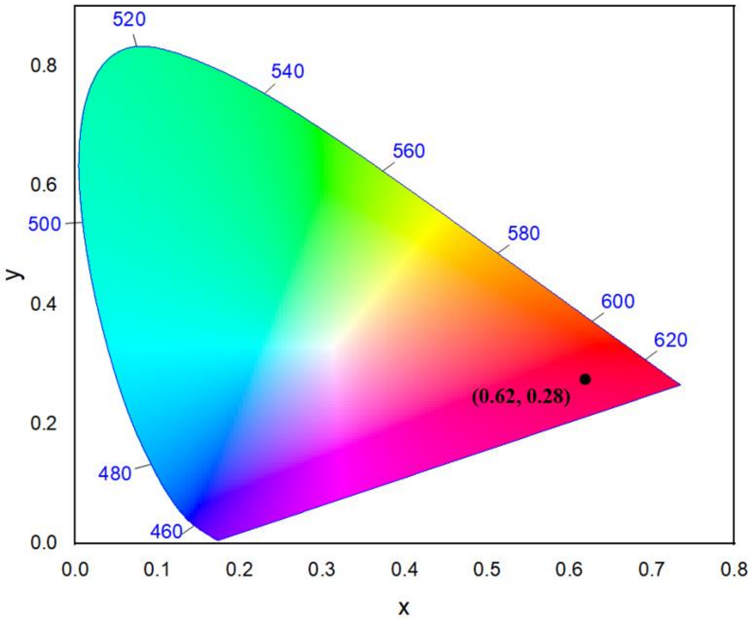

Fig. S9 The Commission Internationale de l'Eclairage (CIE) color coordinates of the GuI treated CsPbI3 NCs LEDs

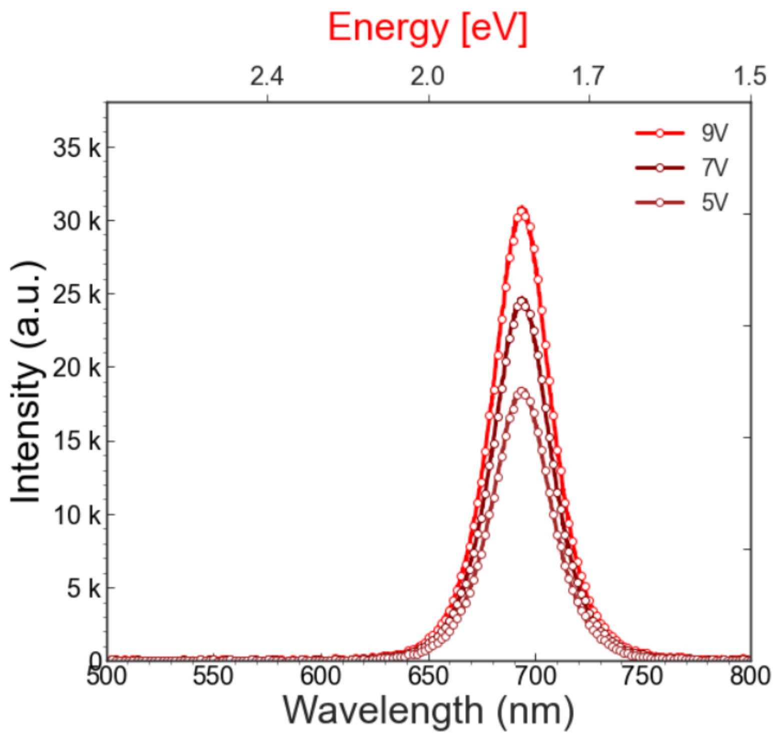

Fig. S10 The EL spectra of LED fabricated from GuI treated $CsPbI_3$ NCs operating at different driving voltages

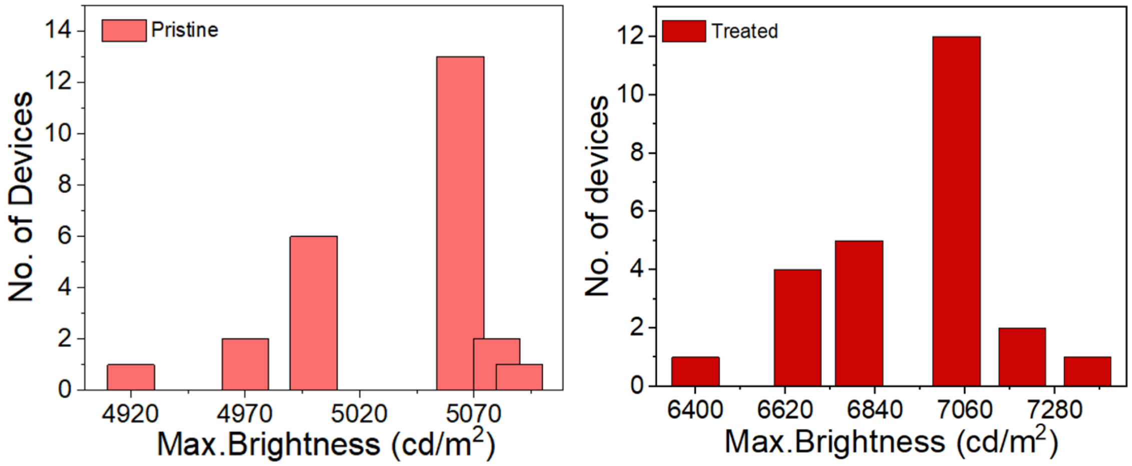

Fig. S11 Histogram of maximum brightness of multiple devices made from pristine (left) and treated (right) $CsPbI_3$ NCs

Table S1 Relative PLQY detail calculation using Rhodamine 6G as reference dye [S1, S2]

$Q_x = Q_R \frac{I_x}{I_R} \frac{A_R}{A_x} \frac{n_x^2}{n_R^2}$	**Absorbance (at 350 nm)**	**Integrated PL intensity**	**FWHM (nm)**	**PLQY (%)**
**Rhodamine 6G**	0.098	27787.82	34.49	95.0
**$CsPbI_3$**	0.101	21572.05	34.96	73.0
**Purified $CsPbI_3$ (Pristine)**	0.099	17143.74	34.25	59.2
**Purified $CsPbI_3$/GuI (Treated)**	0.104	24829.65	36.31	81.6

Table S2 Fitted TR-PL data of pristine CsPbI3 NCs and GuI treated $CsPbI_3$ NCs

Sample	τ_1 (ns)	A_1 (%)	τ_2 (ns)	A_2 (%)	τ_3 (ns)	A_3 (%)	τ_{ave} (ns)
**Pristine**	3.3	13.7	19.8	42.4	76.8	43.9	64.7
**Treated**	25.4	47.7	85.4	52.3	0	0	72.6

Table S3 The table summarized the reported performance of red perovskite LED using different surface treatment method in comparison with our work

Perovskite materials	Device structure	EL peak (nm)	EQE (%)	Maximum brightness (cd m^{-2})	Stability	Year, Refs.
$CsPb(Br/I)_3$ post treated with polyethylenimine	ITO/ZnO/ PEI/ PSK/ CBP/ TCTA/ MoO_3/ Au	648	6.3	2450	NA	2016[S3]
$CsPbI_{3-x}Br_x$ NCs with KBr passivation	ITO/ PEDOT:PSS/ Poly-TPD/ PSK/ TPBi/ LiF/ Al.	637	3.55	2671	T_{50} = 50 min at 5.0 V constant voltage.	2020[S4]
$CsPbI_3$ NCs with benzyl iodide surface treatment	ITO/ PEDOT:PSS/ Poly-TPD/ PSK/ TPBi/ LiF/ Al.	625	12.9	3382	NA	2020[S5]
$CsPbI_3$ NCs with Zirconium Acetylacetonate surface modification	Si/Ag/ZnO/ PEI/ PSK/ TCTA/ MoO_3/ Au	686	13.7	14725	NA	2020[S6]
$CsPbI_3$ NCs with 1-hydroxy-3-phenylpropan-2-aminium iodide (HPAI) and tributylsulfonium iodide (TBSI) post treatment.	ITO/ PEDOT:PSS/ PTAA/PSK/ PO-T2T/ LiF/ Al	630	6.4	1212	T_{50} = 78 min at current density of 1 mA cm^{-2}	2021[S7]
$MAPb(I_{1-x}Br_x)_3$ NCs treated with multidentate ligands	ITO/ PEDOT:PSS/ Poly-TPD/ TFB/ PSK/ TPBi/ LiF/ Al.	620	20.3	627	T_{50} = 340, 130, 16 min at current density of 0.1, 1 and 10 mA cm^{-2}	2021[S8]
$CsPbI_3$ NCs incorporated with poly(maleic anhydride-alt-1-octadecene) (PMA)	ITO/ PEDOT:PSS + PFI/ Poly-TPD/ PSK/ TPBi/ LiF/ Al.	690	17.8	618	T_{50} = 317 hours at current density of 30 mA cm^{-2}	2021[S9]

$CsPbI_3$ NCs passivated with naphthylmethyl-ammonium iodide and incorporated with $CH_3CH_2NH_3I$	ITO/ZnO/ PEI/ PSK/ TCTA/ MoO_3/ Au	694	17.5	403	NA	2021[S10]
$CsPbI_{3-x}Br_x$ NCs with Tetraoctyl-ammonium Bromide post treatment	ITO/ PEDOT:PSS VB-FNPD/ PSK/ TPBi/ LiF/ Al.	667	11.7	1345	NA	2021 (DOI: 10.1016/ j.jallcom.2021. 163182)
$CsPbI_3$ NCs with Zn, Mn doping and KI surface treatment	ITO/ PEDOT:PSS + PFI/ Poly-TPD/ PSK/ TPBi/ LiF/ Al.	640	23	~1500	half-lifetime of 10 h (luminance of 200 cd m^{-2})	2021[S11]
$CsPbI_3$ NCs with GuI surface treatment.	ITO/ PEDOT:PSS PSK/ TPBi/ LiF/ Ag.	695	13.8	7039	T_{50} ~ 20 min at current density of 25 mA cm^{-2}	This work

Supplementary References

[S1] D. Magde, R. Wong, P.G. Seybold, Fluorescence quantum yields and their relation to lifetimes of rhodamine 6G and fluorescein in nine solvents: improved absolute standards for quantum yields. Photochem. Photobiol. **75**(4), 327-334 (2002). https://doi.org/10.1562/0031-8655(2002)0750327FQYATR2.0.CO2

[S2] G.A. Crosby, J.N. Demas, Measurement of photoluminescence quantum yields. Rev. J. Phys. Chem. **75**(8), 991-1024 (1971). https://doi.org/10.1021/j100678a001

[S3] X. Zhang, C. Sun, Y. Zhang, H. Wu, C. Ji et al., Bright perovskite nanocrystal films for efficient light-emitting devices. J. Phys. Chem. Lett. **7**(22), 4602-4610 (2016). https://doi.org/10.1021/acs.jpclett.6b02073

[S4] J.N. Yang, Y. Song, J.S. Yao, K.H. Wang, J.J. Wang et al., Potassium bromide surface passivation on $CsPbI_{3-x}Br_x$ nanocrystals for efficient and stable pure red perovskite light emitting diodes. J. Am. Chem. Soc. **142**(6), 2956-2967 (2020). https://doi.org/10.1021/jacs.9b11719

[S5] H. Wang, Y. Dou, P. Shen, L. Kong, H. Yuan et al., Molecule-induced p-doping in perovskite nanocrystals enables efficient color-saturated red light-emitting diodes. Small **16**(20), 2001062 (2020). https://doi.org/10.1002/smll.202001062

[S6] M. Lu, J. Guo, S. Sun, P. Lu, J. Wu et al., Bright $CsPbI_3$ perovskite quantum dot light-emitting diodes with top-emitting structure and a low efficiency roll-off realized by applying zirconium acetylacetonate surface modification. Nano Lett. **20**(4), 2829-2836 (2020). https://doi.org/10.1021/acs.nanolett.0c00545

[S7] Y.F. Lan, J.S. Yao, J.N. Yang, Y.H. Song, X.C. Ru et al., Spectrally stable and efficient pure red $CsPbI_3$ quantum dot light-emitting diodes enabled by sequential ligand post-treatment strategy. Nano Lett. **21**(20), 8756-8763 (2021). https://doi.org/10.1021/acs.nanolett.1c03011

[S8] Y. Hassan, J.H. Park, M.L. Crawford, A. Sadhanala J.C. Sadighian, et al., Ligand-engineered bandgap stability in mixed-halide perovskite LEDs. Nature **591**, 72-77 (2021). https://doi.org/10.1038/s41586-021-03217-8

[S9] H. Li, H. Lin, D. Ouyang, C. Yao, C. Li et al., Efficient and stable red perovskite light-emitting diodes with operational stability>300 h. Adv. Mater. **33**(15), 2008820 (2021). https://doi.org/10.1002/adma.202008820

[S10] G. Sun, X. Liu, Z. Liu, D. Liu, F. Meng et al., Emission wavelength tuning via competing lattice expansion and octahedral tilting for efficient red perovskite light-emitting diodes. Adv. Funct. Mater. **31**(50), 2106691 (2021). https://doi.org/10.1002/adfm.202106691

[S11] Y.K. Wang, F. Yuan, Y. Dong, J.Y. Li, A. Johnston et al., All-inorganic quantum dot LEDs based on phase-stabilized α-$CsPbI_3$ perovskite. Angew. Chem. Int. Ed. **60**(29), 16164-16170 (2021). https://doi.org/10.1002/anie.202104812
